# Functional parameters and affecting factors in post-COVID period

**DOI:** 10.5578/tt.20239915

**Published:** 2023-06-13

**Authors:** D. KIZILIRMAKR, U. FİDAN, S. SARI, Y. HAVLUCU

**Affiliations:** 1 Manisa Celal Bayar University Faculty of Medicine, Department of Chest Diseases, Manisa, Türkiye; 1 Therapist Celal Bayar University Manisa Hafsa Sultan Hospital, Respiratory Manisa, Türkiye

**Keywords:** Post-COVID, 6-minute walk test, functional status, Post-COVID, 6 dakika yürüme testi, fonksiyonel durum

## Abstract

**ABSTRACT:**

Functional parameters and affecting factors in post-COVID period

**Introduction:**

Post-COVID period is considered to be 12 weeks after the COVID-19 infection. Patients in the post-COVID period may have prolonged or newly developed symptoms. Depending on the prolonged effects of the disease, respiratory and functional parameters may be affected. The aim of the study is to investigate the effect of COVID-19 infection on respiratory and functional parameters in the post-COVID period.

**Materials and Methods:**

A cross-sectional study was conducted to evaluate the functional parameters of patients with COVID-19 in the post-COVID period. Subjects with a history of microbiologically proven COVID-19 infection were evaluated with 6-minute walk test results, Borg, and MRC results at least 12 weeks after COVID-19 infection. The relationship between demographic characteristics, comorbidities, vaccination status, and severity of disease with 6-minute walk test results and dyspnea scales in the post-COVID period was investigated.

**Results:**

Two hundred seventeen patients were included in the study. The mean age of the patients was 48.6 ± 14.9 years and 126 (58.1%) of them were female. 142 (65.4%) of the patients were completely vaccinated against COVID-19 and 75 (34.6%) patients were incompletely vaccinated or unvaccinated. 158 (72.8%) patients had mild disease, 51 (23.5%) patients had moderate disease, and eight (3.7%) patients had severe disease. Those with a history of moderate or severe disease had significantly worsened functional parameters in the postCOVID period compared to those with mild COVID-19. The Borg scale and MRC dyspnea scale values were significantly higher in women (p= 0.008, p= 0.002, respectively). Functional parameters of those who were completely vaccinated against COVID-19 and those who were incompletely or unvaccinated individuals in the post-COVID period were similar.

**Conclusion:**

The functional parameters of people with moderate or severe COVID-19 disease were found to be significantly impaired in the post-COVID period. While the effect of smoking and vaccination status on functional parameters in the post-COVID period could not be demonstrated, disease severity and accompanying comorbidity were found to be effective.

## Abstract (tr)

**ÖZ:**

Post-COVID dönemde fonksiyonel parametreler ve etkileyen faktörler

**Giriş:**

COVID-19 enfeksiyonundan 12 hafta sonraki dönem post-COVID dönem olarak kabul edilmektedir. Post-COVID dönemdeki hastalarda uzamış veya yeni gelişmiş semptomlar görülebilmektedir. Hastalığın uzamış etkilerine bağlı olarak solunumsal ve fonksiyonel parametreler etkilenebilmektedir. Bu çalışmada, COVID-19 enfeksiyonunun post-COVID dönemdeki solunumsal ve fonksiyonel parametrelere etkisinin araştırılması amaçlanmıştır.

**Materyal ve Metod:**

COVID-19 geçiren hastaların post-COVID dönemdeki fonksiyonel parametrelerini değerlendirmek amacıyla kesitsel bir çalışma yürütüldü. Çalışmada, mikrobiyolojik olarak kanıtlı COVID-19 enfeksiyonu öyküsü olan kişiler COVID-19 enfeksiyonundan en az 12 hafta sonra 6 dakika yürüme testi sonuçları, Borg ve MRC sonuçlarıyla değerlendirildi. Altı dakika yürüme testi sonrasında ise oksijen satürasyonunun test öncesi değerlere dönme süresi ölçüldü. Demografik özellikler, eşlik eden hastalıklar, COVID-19’a karşı aşılanma durumu ve hastalık ağırlığının Post-COVID dönemde 6 dakika yürüme testi sonuçları ve dispne skalaları ile ilişkisi araştırıldı.

**Bulgular:**

Araştırmaya 217 hasta dahil edilmiştir. Hastaların yaş ortalaması 48,6 ± 14,9’du ve 126’sı (%58,1) kadındı. Yüz kırk iki (%65,4) hasta COVID-19’a karşı tam aşılı, 75 (%34,6) hasta ise eksik aşılı veya aşısızdı. Yüz elli sekiz (%72,8) hasta hafif, 51 (%23,5) hasta orta, sekiz (%3,7) hasta ise ağır hastalık öyküsüne sahipti. Orta veya ağır hastalık öyküsü olanların, hafif COVID-19 geçirenlere göre post-COVID dönemdeki fonksiyonel parametreleri belirgin olarak kötüleşmişti. Kadınlarda Borg skalası ve MRC dispne skalası değerleri anlamlı olarak yüksekti (sırasıyla; p= 0,008, p= 0,002). COVID-19’a karşı tam aşılanmış olanlar ile eksik aşılanmış veya aşılanmamış bireylerin post-COVID dönemdeki fonksiyonel parametreleri benzerdi.

**Sonuç:**

Çalışmamızda, orta veya ağır COVID-19 hastalığı geçiren kişilerin post-COVID dönemdeki fonksiyonel parametrelerinin belirgin olarak bozulduğu saptanmıştır. Post-COVID dönemdeki fonksiyonel parametreler üzerinde sigara kullanımı ve aşılanma durumu etkili bulunmamış, hastalık ağırlığı ve eşlik eden komorbidite durumu etkili bulunmuştur.

## Introduction

More than 646 million people all over the world have been infected with COVID-19 caused by the SARSCoV-2 virus ( 1 ). A severe course of the disease has been observed in approximately 15% of those infected. In fact, 5% of patients develop acute respiratory failure with or without multi-organ failure. It has been shown that people with chronic diseases and those over the age of 65 are more likely to have severe COVID-19 infection ( 2 ). The vast majority of people with COVID-19 recover after the acute phase. However, some patients may experience ongoing symptoms and findings even after the acute phase.

The term used to describe the persistence of signs and symptoms of COVID-19 for up to four weeks after symptom onset is “acute COVID-19 infection”. The period between the 4th week and the 12th week from the onset of symptoms is named “ongoing symptomatic COVID-19” or “subacute COVID-19”. The period after 12 weeks is named the “post-COVID period” ( 3 ). While individuals recovering from COVID-19 may experience prolonged symptoms, it is also possible for new symptoms to emerge during the post-acute period that were not present during the acute phase of the illness. These symptoms may be due to viral infection, intensive care therapy, vascular damage, organ damage, or various other mechanisms.

A significant number of patients with COVID-19 infection appear to suffer from long-term respiratory dysfunction, residual pulmonary parenchymal abnormalities, reduced physical capacity, loss of muscle mass, anxiety, depression, fatigue, and cognitive dysfunction ( 4 ). Although the respiratory effects and the functional limitations caused by COVID-19 significantly affect the quality of life of the patients, the clinical factors that affect the pulmonary function parameters in the post-COVID period are not clearly known.

This study aimed to assess the functional parameters of patients who sought care at the chest diseases outpatient clinic during the post-COVID period and identify factors that may influence these parameters. The findings from this study can contribute to the development of diagnostic, follow-up, and treatment algorithms for post-COVID cases, as well as provide valuable epidemiological data for healthcare planning purposes.

## MATERIALS and METHODS

Patients with a history of microbiologically proven COVID-19 who were followed up in the post-COVID period in the chest diseases outpatient clinic were included in the study. Patients were evaluated in terms of demographic data, COVID-19-associated data, clinical features, and functional parameters in the post-COVID period. Ethics committee approval (Decision no: 303, date: 25.07.2022) was obtained from the Manisa Celal Bayar University Clinical Research Ethics Committee to conduct the study.

**Figure 1:**
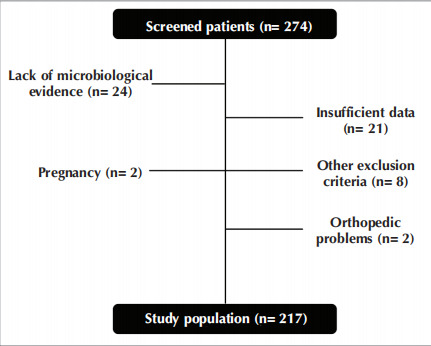
Consort diagram. n: Number.

The inclusion criteria for this study included being aged 18 years or older, having a confirmed microbiological diagnosis of COVID-19, being in the post-positivity period between 12 and 24 weeks, providing informed consent to participate in the study, and being willing to communicate and cooperate with the researchers. Individuals who did not have microbiologically confirmed COVID-19 infection, those with missing or incomplete data, pregnant women, individuals who were unable to cooperate or had insufficient mental capacity for functional evaluation, individuals with active malignancies that could affect the functional evaluation, and those with a history of continuous oxygen therapy prior to COVID-19 infection, systemic rheumatological, endocrinological, and hematological diseases, congestive heart failure, and orthopedic problems that would hinder the walking test were excluded from the study ( Figure 1 ).

Informed consent forms were obtained from all patients included in the study. Demographic data and registration forms were filled face to face. Participants’ age, gender, body mass index (BMI), occupation, smoking status, comorbidities, vaccination status against COVID-19, and severity of COVID-19 disease were questioned. The patients were categorized into groups based on the severity of their acute COVID19 disease status according to the criteria set by the

World Health Organization (WHO). Mild disease was defined as fever, sore throat, cough, headache, joint pain, fatigue-like symptoms without respiratory distress, and pathological radiological findings. Moderate disease was defined as signs and symptoms of respiratory tract infection with oxygen saturation (SO_2_) of 90-94% or above. Severe disease was defined as a respiratory rate of above 30/minute, SO_2_ lower than 90-94%, PaO_2_/FiO_2_ value below 300 mmHg, or involvement in more than 50% of the lungs ( 5 ). Fully vaccinated individuals were defined as those who had received at least two doses and whose last dose was within the last six months. Incomplete vaccination was defined as either receiving a single dose of the vaccine or receiving the last dose more than six months ago. CoronaVac and Comirnaty vaccines, which were used in Türkiye after obtaining emergency use approval, were administered to the patients.

The medical research council (MRC) and Borg dyspnea scales were used to assess the dyspnea grades of the patients. The MRC is a five-item scale based on various activities that cause dyspnea. The MRC scale is graded as; 1- Having shortness of breath during strenuous exercises, 2- Having shortness of breath when going uphill or moving quickly on flat ground, 3- Having to move slowly for their age due to shortness of breath or having to stand while walking at normal speed on flat ground, 4- Having to stop after walking 100 m or a few minutes on flat ground, 5- Being home-bound and having shortness of breath during activities such as getting dressed ( 6 ). The severity of dyspnea before and after the 6-minute walk test (6MWT) was measured with the Borg dyspnea scale. The Borg scale is a ten-item scale used to instantly evaluate the severity of dyspnea at rest and on exertion and defines the severity of dyspnea according to its degrees. The Borg scale is graded as “0- Nothing at all, 0.5- Very very slight, 1- Very slight, 2- Slight, 3- Moderate, 4- Somewhat severe, 5- Severe, 7- Very severe, 9- Very very severe and 10- Maximal” ( 7 ).

All participants underwent 6MWT for objective assessment of cardiopulmonary function. 6MWT allows the integrated evaluation of many systems involved in physical activity. The test is based on the patient walking for 6 minutes in a corridor of a certain length with marked start and end points ( 8 ). Before the test, patients were seated and rested for about 15 minutes, and arterial blood pressure, heart rate, oxygen saturation values, and severity of shortness of breath were recorded. After the test was completed, the patients were seated and rested, the measurements were repeated, and the time for the oxygen saturation to return to pre-test values was measured. The relationships between demographic characteristics, comorbidities, vaccination status against COVID-19, and severity of disease with 6MWT results and dyspnea scales in the postCOVID period were investigated.

• The data obtained in the study were statistically evaluated with the “IBM SPSS Statistics 23” software. Frequency, percentage values, median (interquartile range), mean and standard deviation values were determined as descriptive statistics. Numerical variables in the comparisons were in a normal distribution. An Independent sample t-test was used for these variables. Comparisons between categorical variables were evaluated with the Chi-square test. Statistical results were investigated by linear regression analysis for potential confounders. In these statistical calculations, p< 0.05 was considered statistically significant.

## RESULTS

A total of 217 patients followed in the post-COVID period were included in the study. The mean age of the patients was 48.6 ± 14.9. 126 (58.1%) patients were female and 91 (41.9%) were male. The mean body mass index of all patients was 27.7 ± 5.5 kg/ m^2^. Seventy-six (35.0%) were smokers or had smoked before, and 141 (65.0%) had never smoked. The average time from COVID-19 positivity to the date when patients were evaluated was 16 weeks and three days. The most common comorbidities were hypertension, diabetes, and asthma, respectively. Of the patients, 46 (21.2%) had hypertension, 39 (18%) had diabetes, 23 (10.6%) had asthma, 21 (9.7%) had coronary artery disease, seven (3.2%) had chronic obstructive pulmonary disease and four (1.8%) had parenchymal lung disease. Two patients with parenchymal lung disease had idiopathic pulmonary fibrosis and two had lung involvement of connective tissue disease.

When the participants were examined in terms of their immunization status against COVID-19, 142 (65.4%) patients were completely vaccinated, 18 (8.3%) patients were vaccinated with a missing dose while 57 (26.3%) patients were not vaccinated against COVID-19. 158 (72.8%) patients had mild, 51 (23.5%) patients had moderate, and eight (3.7%) patients had severe disease history. Nine (4.1%) patients needed long-term oxygen therapy at home after COVID-19 treatment. The demographics and clinical characteristics of the patients are presented in Table 1.

**Table 1:** Demographics and clinical characteristics

Parameters	MIP< -80 cm H2O n= 58	MIP≥ -80 cm H2O n= 28	Total	P*
Demographic Features				

*The t-test or Mann-Whitney U test was used for the comparisons of groups Variables were given as mean ± SD and median (min:max). MIP: Maximum inspiratory pressure, MEP: Maximum expiratory pressure, MRC: Medical Research Council, FVC: Forced vital capacity, FEV1: Forced expiratory volume in one second, BMI: Body mass index, FFMI: Fat- free mass index, ISWT: Incremental shuttle walking test, ESWT: Endurance shuttle walking test, SGRQ: St. George’s Respiratory Questionnaire, CRQ: Chronic respiratory questionnaire.

• In the post-COVID period, the 6MWT distances of both women and men were statistically similar. While the mean 6MWT distance for women was 409.68 ± 119.86 meters, it was 428.79 ± 130.44 meters for men (p= 0.27). However, the oxygen saturation at rest and after 6MWT, and the recovery time for oxygen saturation were significantly better in women. Resting oxygen saturation was 97.52 ± 2.21% in women and 96.27 ± 2.75% in men (p< 0.001). Oxygen saturation after 6MWT was 94.80 ± 3.70% in women and 93.44 ± 4.08% in men (p= 0.011). The recovery time of oxygen saturation to resting level after 6MWT was 9.33 ± 18.56 seconds in women and 14.73 ± 20.79 in men (p= 0.046). Contrary to functional parameters, dyspnea perception was higher in women than in men. While the mean MRC score was 3.36 ± 1.00 in women, it was 2.86 ± 1.30 in men (p= 0.002). The mean Borg values at rest and after 6MWT were 2.15 ± 1.09 and 3.90 ± 1.96 in women, while it was 1.86 ± 1.07 and 3.16 ± 2.00 in men (respectively; p= 0.049, p= 0.008).

• There were no significant differences between the immunization status of the participants against COVID-19 and the functional outcomes in the post-COVID period. The mean 6MWT distance of 75 (34.6%) patients who were unvaccinated or incompletely vaccinated against COVID19 was 413.87 ± 117.17 meters, while it was 419.72 ± 128.52 meters for 142 (65.4%) fully vaccinated patients (p= 0.74). Oxygen saturation at rest and after 6MWT was 97.15 ± 1.84% and 94.74 ± 3.07% in unvaccinated or incompletely vaccinated patients, however, it was measured as 96.92 ± 2.82% and 93.96% ± 4.28% in fully vaccinated patients (respectively; p= 0.52, p= 0.16). The recovery time of oxygen saturation to resting level after 6MWT was 8.65 ± 15.48 seconds in unvaccinated or incompletely vaccinated patients and 13.15 ± 21.43 seconds in fully vaccinated patients (p= 0.11). When comparing patients based on smoking status, no significant differences were observed in terms of functional parameters and respiratory symptoms between smokers and non-smokers. Those with chronic lung disease, diabetes, and hypertension had significantly worse 6-minute walking test distances (respectively; p= 0.047, p= 0.019, p< 0.001).

• The mean 6MWT walking distance of 158 (72.8%) patients with mild disease was 439.4 ± 108.6 meters, and of 59 (27.2%) patients with moderate or severe disease was 359.7 ± 145.2 meters. Resting oxygen saturation values measured before 6MWT were 97.37 ± 2.26% in patients with mild disease and 95.98 ± 2.89% in those with moderate or severe disease. Oxygen saturation values measured after 6MWT were 94.58 ± 3.67% in mild disease and 93.31 ± 4.40% in moderate or severe disease. Oxygen saturation recovery time after 6MWT was 10.65 ± 18.56 seconds in patients with mild disease and 14.12 ± 22.33 seconds in patients with moderate or severe disease. Resting Borg values were 1.91 ± 0.98 in patients with mild disease and 2.36 ± 1.30 in patients with moderate or severe disease. Borg values after 6MWT were 3.50 ± 1.86 in the mild disease group and 3.83 ± 2.34 in the moderate or severe disease group. The mean MRC scale value was 3.03 ± 1.12 in the mild disease group and 3.46 ± 1.21 in the moderate or severe disease group. When the results were evaluated by regression analysis in terms of age, gender, BMI, smoking status, and vaccination status, 6MWT messages were longer and resting oxygen saturation was higher in mild disease group. The relationship between disease severity and functional parameters is presented in Table 2.

The mean age of patients completely vaccinated against COVID-19 and incomplete or unvaccinated patients were similar. The mean age of the subjects who were vaccinated incompletely or unvaccinated was 46.71 ± 15.26, and the mean age of the subjects who were fully vaccinated was 49.60 ± 14.60 (p= 0.17). The vaccination habits of men and women were also similar. 82 of 126 female patients and 60 of 91 male patients were fully vaccinated (p= 0.99). Of the 75 incomplete vaccinated or unvaccinated patients, 48 had mild disease and 27 had moderate or severe disease. This situation was significantly better in 142 fully vaccinated individuals, 110 with mild disease, and 32 with moderate or severe disease (p= 0.038). When evaluated by regression analysis in terms of age, gender, BMI, and smoking status, patients who were fully vaccinated had a milder disease history (p= 0.008) ( Table 3).

**Table 2:** Demographics and clinical characteristics

Parameters	MIP< -80 cm H2O n= 58	MIP≥ -80 cm H2O n= 28	Total	P*
Demographic Features				

*The t-test or Mann-Whitney U test was used for the comparisons of groups Variables were given as mean ± SD and median (min:max). MIP: Maximum inspiratory pressure, MEP: Maximum expiratory pressure, MRC: Medical Research Council, FVC: Forced vital capacity, FEV1: Forced expiratory volume in one second, BMI: Body mass index, FFMI: Fat- free mass index, ISWT: Incremental shuttle walking test, ESWT: Endurance shuttle walking test, SGRQ: St. George’s Respiratory Questionnaire, CRQ: Chronic respiratory questionnaire.

**Table 3:** Demographics and clinical characteristics

Parameters	MIP< -80 cm H2O n= 58	MIP≥ -80 cm H2O n= 28	Total	P*
Demographic Features				

*The t-test or Mann-Whitney U test was used for the comparisons of groups Variables were given as mean ± SD and median (min:max). MIP: Maximum inspiratory pressure, MEP: Maximum expiratory pressure, MRC: Medical Research Council, FVC: Forced vital capacity, FEV1: Forced expiratory volume in one second, BMI: Body mass index, FFMI: Fat- free mass index, ISWT: Incremental shuttle walking test, ESWT: Endurance shuttle walking test, SGRQ: St. George’s Respiratory Questionnaire, CRQ: Chronic respiratory questionnaire.

## DISCUSSION

Respiratory and functional parameters may be affected due to the prolonged effects of COVID-19. In our study, it was determined that the functional parameters of people with a history of moderate or severe COVID-19 disease were significantly affected in the post-COVID period. In people with chronic lung disease, diabetes, and hypertension, functional parameters were found to be significantly impaired in the post-COVID period. However, the relationship between vaccination status and smoking status with functional parameters in the post-COVID period could not be demonstrated.

Although data on the long-term effects of COVID-19 are still limited, our results show similarities with other studies in the literature. Chronic complaints and poor quality of life are observed in a significant proportion of patients who have recovered from COVID-19 infection. In the study of Carfi et al., it was observed that at least one symptom (most frequently dyspnea and fatigue) persisted in 87% of the patients in the post-COVID period ( 9 ). Garrigues et al. found persistent respiratory symptoms and deterioration in quality of life in the post-COVID period in patients requiring hospitalization and intensive care follow-up ( 10 ). Approximately half of the patients who recovered from the COVID-19 infection report shortness of breath 2-3 months after the infection ( 11 ). Functional parameters in the post-COVID period were found to be associated with severe disease, similar to our study. In the post-COVID period, 6MWT and oxygen saturation values were found to be worse in people with a long history of hospitalization in the intensive care unit ( 12 ).

In our study, although the 6MWT distances of male and female patients in the post-COVID period were similar, the resting and post-6MWT oxygen saturation and recovery time were significantly worse in men. However, dyspnea symptoms were significantly more common in women. Unlike our study, a study found significantly worse 6MWT and pulmonary function test results in the post-COVID period in women who had COVID-19 pneumonia (13). Respiratory symptoms of women in the post-COVID period were worse than men in other studies in the literature, similar to our study ( 14 , 15 , 16 ).

There are many studies showing the relationship between vaccination against COVID-19 and disease severity. In particular, mRNA vaccines significantly reduce hospitalization, the need for intubation, and mortality of the disease ( 17 ). In our study, although vaccination against COVID-19 was found to be associated with disease severity, the relationship between vaccination and functional parameters in the postCOVID period could not be demonstrated. Although there are a few studies in the literature showing that vaccination against COVID-19 reduces respiratory symptoms in the post-COVID period, there are no studies investigating the effects of vaccination status on functional parameters in the post-COVID period ( 18 , 19 ).

The complexity and variability of the damage caused by COVID-19 and the accompanying comorbidities of many patients with COVID-19 make it difficult to control symptoms and treatment in the post-COVID period (20). Conditions such as shortness of breath, cough, impaired exercise capacity, fatigue, debility, weakening in cognitive functions, and muscle weakness in the post-COVID period cause increased hospital admissions ( 21 ). Informing by physicians has a great effect on reducing healthcare provider applications. In particular, patients with a history of moderate or severe disease and accompanying comorbidities should be followed up in terms of functional limitations in the post-COVID period and evaluated for treatment. In the post-COVID period, rehabilitation is beneficial in improving the respiratory functions of patients, eliminating immobility, preventing long-term complications and disability, increasing cognitive and emotional well-being, and increasing the quality of life and independence in daily activities ( 22 , 23 ). Patients with functional limitations in the post-COVID period may benefit from early rehabilitation modalities.

The main limitations of our study are the unknown condition of patients before COVID-19 infection, the under-representation of the elderly population, the inability to evaluate comorbidities that may affect functional parameters in detail, and the availability of data from a single center. The fact that the patients included in the study were selected among the patients who applied to the post-COVID outpatient clinic may have caused the respiratory symptoms and functional outcomes to be worse than the general population. These negativities could not be avoided due to the design of the study, the inability to detect people who will have COVID-19 in advance, and the inability to exclude confounding factors completely

The outstanding aspects of our study are that although it is a single-center study, a sufficient number of patients could be evaluated in detail in the postCOVID period, and all 6MWTs and dyspnea scales were administered by a single person without knowledge of the research hypothesis. There are few studies in the literature evaluating this patient group in detail. With these aspects, this study provides important data investigating the factors affecting functional parameters in the post-COVID period. There is a need for multicenter studies in which functional status before COVID-19 infection and confounding factors can be investigated.

## CONCLUSION

Significant deterioration in functional parameters was observed in individuals with moderate or severe COVID-19 infection and those with cardiopulmonary comorbidities during the post-COVID period. It is recommended to closely monitor these individuals for functional limitations, treatment needs, and pulmonary rehabilitation. While milder disease was observed in vaccinated individuals, the impact of vaccination and smoking status on functional parameters during the post-COVID period could not be determined. Studying the long-term effects and contributing factors of COVID-19, a global viral infection that has caused the largest pandemic of our century, will provide valuable insights into potential similar situations that may arise in the future.

## Ethical Committee Approval

The study was approved by Manisa Celal Bayar University Faculty of Medicine Clinical Research Ethics Committee (Decision no: 303, Date: 25.07.2022).

## Conflict of INTEREST

The authors declare that they have no conflict of interest.

## AUTHORSHIP CONTRIBUTIONS

Concept/Design: DK, YH, UF

Analysis/Interpretation: DK, UF, SS

Data acqusition: : SS, UF

Writing: DK, UF, YH

Clinical Revision: SS, YH, DK

Final Approval: All of authors
